# Carbapenem-Resistant *Enterobacter* *cloacae* in Patients from the US Veterans Health Administration, 2006–2015

**DOI:** 10.3201/eid2305.162034

**Published:** 2017-05

**Authors:** Brigid M. Wilson, Nadim G. El Chakhtoura, Sachin Patel, Elie Saade, Curtis J. Donskey, Robert A. Bonomo, Federico Perez

**Affiliations:** Louis Stokes Cleveland Department of Veterans Affairs Medical Center, Cleveland, Ohio, USA (B.M. Wilson, N.G. El Chakhtoura, C.J. Donskey, R.A. Bonomo, F. Perez);; University Hospitals Cleveland Medical Center, Cleveland (N.G. El Chakhtoura, S. Patel, E. Saade, R.A. Bonomo);; Case Western Reserve University School of Medicine, Cleveland (C.J. Donskey, R.A. Bonomo, F. Perez)

**Keywords:** carbapenem resistance, antimicrobial resistance, Enterobacteriaceae, Enterobacter cloacae complex, Klebsiella pneumoniae, carbapenemase, United States, US Veterans Health Administration, bacteria

## Abstract

We analyzed carbapenem-resistant *Enterobacteriaceae* (CRE) trends among patients from the US Veterans Health Administration (VHA). After the emergence of CRE in the eastern United States, resistance rates remained stable in *Klebsiella pneumoniae* but increased in *Enterobacter cloacae* complex, suggesting a "second epidemic". VHA offers a vantage point for monitoring nationwide CRE trends.

Carbapenem-resistant *Enterobacteriaceae* (CRE) have become a global public health threat. The epidemic of CRE began in the early 2000s with an outbreak of carbapenem-resistant *Klebsiella pneumoniae* harboring *K. pneumoniae* carbapenemase (KPC) in the eastern United States. Since then, KPC-producing *K. pneumoniae* have emerged in various communities across the country (*1*). Carbapenem resistance also occurs in other *Enterobacteriaceae* species and can be mediated by other enzymes, such as OXA-48 and metallo-β-lactamases, especially New Delhi metallo-β-lactamase and Verona integron–encoded metallo-β-lactamase (*1*). Carbapenem-resistant *Escherichia coli* occurs infrequently, but recent outbreaks of KPC-producing *Enterobacter cloacae* raise concerns about the emergence of carbapenem resistance in the *E. cloacae* complex (*1*–*4*).

The Veterans Health Administration (VHA) is the largest integrated healthcare system in the United States. Clinical and microbiologic data for the entire VHA network are accessible through its informatics platforms (*5*). We used this infrastructure to observe national trends of carbapenem resistance and nonsusceptibility in *K. pneumoniae* and *E. cloacae* complex during the past decade.

We identified 224,651 *K. pneumoniae* and 71,462 *E. cloacae* complex (*E. cloacae, E. asburiae, E. kobei, E. hormaechei, E. xiafangensis*) isolates from patients hospitalized during 2006–2015. To minimize bias introduced by variability in susceptiblity reporting, we excluded isolates obtained within 30 days of another isolate from the same patient and isolates from facilities with nonstandard reporting, facilitites that identified <30 isolates in a 2-year period, and facilities that reported carbapenem susceptibilities for <90% of isolates. After these exclusions, 128,431 *K. pneumoniae* and 38,219 *E. cloacae* complex isolates from 140 facilities in 40 states, District of Columbia, and Puerto Rico remained for study. We obtained carbapenem (i.e., meropenem, imipenem, ertapenem, doripenem) susceptibility test results (i.e., susceptible, intermediate, or resistant) and calculated rates of resistance and nonsusceptibility to any carbapenem over time, looking at 2-year windows and grouping facilities into 10 regions designated by the US Department of Health and Human Services (https://www.hhs.gov/about/agencies/regional-offices/index.html).

Our data capture the chronologic and geographic spread of carbapenem-resistant *K. pneumoniae* and *E. cloacae* complex within VHA (Figure). Before 2010, carbapenem-resistant *K. pneumoniae* was observed primarily in the eastern United States, but by 2014–2015, the rate of carbapenem resistance detected in *K. pneumoniae* was >1% in all regions except Regions 8 (South Dakota, North Dakota, Montana, Wyoming, Colorado, Utah) and 10 (Washington, Oregon, Idaho, Alaska). In 2006–2007, carbapenem-resistant *E. cloacae* complex also had a focus in the East, but in 2008–2009, it was also observed in Region 8. By 2014–2015, carbapenem-resistant *E. cloacae* complex was centered in the Southwest and Pacific Coast. These regions also had higher rates of carbapenem-nonsusceptible *E. cloacae* complex, although this phenotype was present in all regions.

**Figure:**
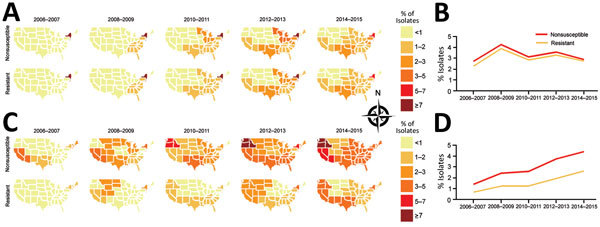
Geographic and temporal trends of carbapenem nonsusceptibility and resistance in *Enterobacteriaceae* seen at Veterans Health Administration facilities, United States, 2006–2015. A) Dissemination of carbapenem-resistant *Klebsiella pneumoniae* after an initial focus in the eastern United States. B) Nationwide percentage of carbapenem nonsusceptibility and resistance in *K. pneumoniae*. C) Emergence and dissemination ("second epidemic") of carbapenem-nonsusceptible and -resistant *Enterobacter cloacae* complex. D) Nationwide percentage of carbapenem nonsusceptibility and resistance in *E. cloacae* complex. Isolates from patients in Puerto Rico were not included in the maps.

The carbapenem-nonsusceptibility rate among *K. pneumoniae* isolates remained at 3%–4% throughout 2006–2015; however, as with a 2016 study (*6*), we detected a recent decrease in the rate of carbapenem resistance in *K. pneumoniae* in Region 2, which includes New York. In contrast, the rates of carbapenem resistance and nonsusceptibility in *E. cloacae* complex increased nationwide during the last decade, reaching >4% nonsusceptibility and 2.5% resistance in 2014–2015. Also, the proportion of intermediate carbapenem-nonsusceptible isolates was higher for *E. cloacae* complex (46%) than *K. pneumoniae* (9%) isolates.

CRE trends during 2006–2015 in the VHA recapitulate the epidemic of carbapenem-resistant *K. pneumoniae* in the United States and indicate that a “second epidemic” of carbapenem-resistant *E. cloacae* complex appears to be unfolding. In the United States, the predominant carbapenem-resistant *K. pneumoniae* genotype is sequence type (ST) 258, which is associated with Tn*4401*, a mobile genetic element containing *bla*_KPC_ (*7*). In contrast, the genetic background of carbapenem-resistant *E. cloacae* complex is not well defined. Analysis of carbapenem-resistant *E. cloacae* from the US Midwest and New York, NY, demonstrated dissemination of *E. cloacae* complex ST171 harboring the *bla*_KPC-3_ gene (*2**,**3**,**8*). Further analysis demonstrated that ST171 was associated with a Tn*4401* variant within a pBK30683-like plasmid; however, various other plasmids in *Enterobacter* spp. also harbor *bla*_KPC-3_ (*4*). Of note, in a northeastern US hospital, one third of carbapenem-resistant *E. cloacae* contained carbapenemases and the rest harbored cephalosporinases, usually only AmpC (*9*). Nevertheless, we hypothesize that *E. cloacae* complex contains genotypes with epidemic potential associated with increasing rates of carbapenem resistance observed in the VHA.

The scope of this study did not include molecular characterization, so we could not determine emerging genotypes or detect outbreaks at individual facilities. Also, nonuniform susceptibility testing and interpretation throughout the VHA may affect reporting of CRE. Although criteria for interpretation of carbapenem susceptibility changed during the past decade, the revised breakpoints do not appear to have a major effect on resistance rates in *Klebsiella* and *Enterobacter* spp., according to other surveillance data (*10*). Despite these limitations, the VHA may serve as a vantage point for detecting nationwide trends in antimicrobial drug resistance. Integration of susceptibility testing with molecular characterization at the VHA may help elucidate the changing epidemiology of CRE in the United States.
